# Allele-specific expression of Parkinson’s disease susceptibility genes in human brain

**DOI:** 10.1038/s41598-020-79990-9

**Published:** 2021-01-12

**Authors:** Margrete Langmyhr, Sandra Pilar Henriksen, Chiara Cappelletti, Wilma D. J. van de Berg, Lasse Pihlstrøm, Mathias Toft

**Affiliations:** 1 Department of Neurology, Oslo University Hospital, Nydalen, P.O. Box 4956, 0424 Oslo, Norway; 2 Institute of Clinical Medicine, University of Oslo, Oslo, Norway; 3 Department of Mechanical, Electronic and Chemical Engineering, OsloMet – Oslo Metropolitan University, Oslo, Norway; 4 Department of Anatomy and Neurosciences, Section Clinical Neuroanatomy and Biobanking, Amsterdam Neuroscience, Amsterdam UMC, Location VU Medical Center, Amsterdam, The Netherlands

**Keywords:** Parkinson's disease, Genome-wide association studies, Quantitative trait

## Abstract

Genome-wide association studies have identified genetic variation in genomic loci associated with susceptibility to Parkinson’s disease (PD), the most common neurodegenerative movement disorder worldwide. We used allelic expression profiling of genes located within PD-associated loci to identify *cis*-regulatory variation affecting gene expression. DNA and RNA were extracted from post-mortem superior frontal gyrus tissue and whole blood samples from PD patients and controls. The relative allelic expression of transcribed SNPs in 12 GWAS risk genes was analysed by real-time qPCR. Allele-specific expression was identified for 9 out of 12 genes tested (*GBA*, *TMEM175*, *RAB7L1*, *NUCKS1*, *MCCC1*, *BCKDK*, *ZNF646*, *LZTS3*, and *WDHD1*) in brain tissue samples. Three genes (*GPNMB*, *STK39* and *SIPA1L2*) did not show significant allele-specific effects. Allele-specific effects were confirmed in whole blood for three genes (*BCKDK*, *LZTS3* and *MCCC1*), whereas two genes (*RAB7L1* and *NUCKS1*) showed brain-specific allelic expression. Our study supports the hypothesis that changes to the *cis*-regulation of gene expression is a major mechanism behind a large proportion of genetic associations in PD. Interestingly, allele-specific expression was also observed for coding variants believed to be causal variants (*GBA* and *TMEM175*), indicating that splicing and other regulatory mechanisms may be involved in disease development.

## Introduction

Variants altering gene expression play a critical role in human health and disease and may be particularly important in neurological disorders as small changes in the gene expression of neurons may affect disease risk^1,2^. A frequent disorder of the brain is Parkinson’s disease (PD), a neurodegenerative movement disorder affecting approximately 1% of the world’s population over 60 years of age. The majority of PD patients are considered to have sporadic disease, likely caused by the cumulative effects of common or rare risk factors, each with a small increase in risk for PD development^3^. Recent genome-wide association studies (GWAS) have discovered a large number of genetic loci, mostly single nucleotide polymorphisms (SNPs), associated with increased risk for PD^4–6^. Although some studies have suggested causal mechanisms for PD risk variants^7^, the functional genes responsible for most of the susceptibility loci remain unknown.

Follow-up analyses of GWAS susceptibility loci aim to establish causal mechanisms underlying the identified associated genetic variants. This is not a straight-forward task since the identified loci normally span numerous genes as a result of the complicated local linkage disequilibrium (LD) structure of many human chromosomal loci. Furthermore, most susceptibility variants map to non-coding regions of the genome, suggesting that the variants may affect the trait through alterations of gene regulation^8–10^. GWAS loci typically contain several regulatory elements that may affect genes at some distance^11,12^. Consequently, there is still much to learn regarding the precise biological mechanisms underlying GWAS associations^13^.

One approach to link functional relevance to trait-associated SNPs in GWAS is to identify genotypes that correlate significantly with the expression level of a gene, so called expression quantitative trait loci (eQTLs)^14,15^. It has been reported that SNPs associated with complex diseases are more likely to be eQTLs than other SNPs^16^. Allelic expression profiling provides a direct way to measure the effect of *cis*-regulatory variation on gene expression. By measuring transcripts from each allele of a gene using a transcribed SNP as a marker to differentiate between the two mRNA copies of heterozygous individuals, the effect of *trans*-acting factors on gene expression is essentially removed since the output from one allele serves as a within-sample control for the other^17^. When allele-specific expression is detected, it indicates the presence of *cis*-regulatory variation in high LD with the transcribed marker used to differentiate between alleles^18^.

We determined the allele-specific expression of genes located in PD-associated loci in the brain and whole blood of PD patients and age-matched controls to gain insight into disease-specific molecular mechanisms. We validated the sensitivity of real-time quantitative PCR (qPCR) to detect allele-specific expression effects and analysed gene expression of PD-associated GWAS loci in post-mortem human brain. By pinpointing genes with allele-specific expression, we highlight some of the genes likely to be involved in disease pathogenesis. Interestingly, we also report that transcribed lead variants identified in GWAS are also eQTLs, suggesting that the molecular mechanism modulating disease risk of such variants may affect splicing or other regulatory functions in addition to amino acid changes in the protein structure as often expected.

## Results

### Identifying transcribed variants for allelic expression analysis of Parkinson’s disease-associated risk loci

**Figure 1:**
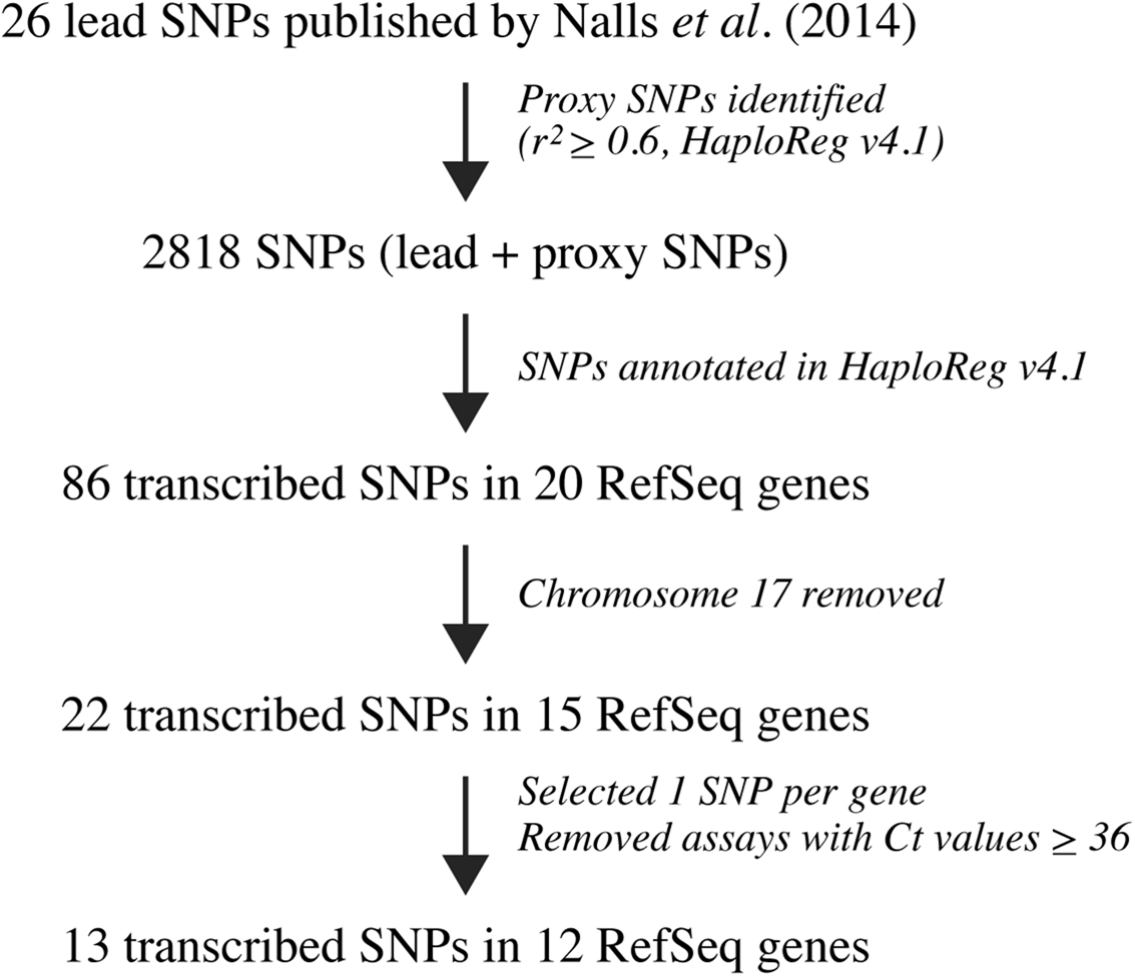
Study design and systematic identification of transcribed single nucleotide polymorphisms (SNPs) suitable for allelic expression analysis of Parkinson’s disease (PD) risk loci. PD genome-wide associated risk SNPs were taken from Nalls et al.^5^ and proxy SNPs were identified in the HaploReg database v4.1. Adapted from Locke et al.^19^. *Ct* cycle threshold.

In 2014, a large-scale meta-analysis of PD GWAS identified and verified 26 genomic loci associated with an increased risk of developing the disease^5^. To analyse the allelic expression of disease-associated risk variants, we selected transcribed variants to compare the relative expression of the two alleles in heterozygous individuals as outlined in Fig. 1. In brief, 2792 proxy SNPs were identified in the Broad Institute HaploReg v4.1 catalogue (last accessed December 2019), applying an LD cut-off threshold of r^2^ ≥ 0.6. Out of the total number of lead and proxy SNPs, 86 out of 2,818 (3.1%) map to transcribed regions of 20 human RefSeq genes. Two loci, rs14235 in *BCKDK* and rs34311866 in *TMEM175*, had coding lead SNPs. We also included the coding secondary signal rs34884217 in *TMEM175*, whereas rs17649553 and its transcribed proxy SNPs in five genes were excluded from further analysis due to their location within a highly complex polymorphic inversion locus on chromosome 17q21. We selected one transcribed SNP per locus and excluded assays where > 50% of the cDNA samples yielded Ct values > 36 (rs2273596 in *TMEM229B*), as this indicates a very low level of gene expression^19^. We excluded the *SNCA* locus because the only transcribed proxy SNP in high LD with lead SNP rs356182, rs356165 (r^2^ = 0.76), is located in the 3′-UTR of *SNCA*. *SNCA* transcripts have variable 3′-UTR lengths and rs356165 is only present in a transcript isoform that contains an extended 3′-UTR region^20^. A rs356165 assay would therefore not be a representative assay to detect allele-specific expression for total *SNCA* mRNA levels, but for a sub-population. Based on these inclusion criteria, allelic expression could be determined for 13 variants in 12 selected genes (Supplementary Fig. S1). The transcribed proxy SNPs that we selected act as markers for allele-specific expression and are not the only candidate causal SNPs in the locus. Table 1 lists all the included SNPs with information on the GWAS-associated gene, the functional annotation of the location of the transcribed proxy SNP and the degree of LD between the transcribed proxy SNP and the lead SNP.

**Table 1:** Lead and transcribed, proxy SNPs selected for allelic expression analysis.

Lead SNP	Transcribed SNP	Gene	Functional annotation	r^2^ (*D*′)	Technology
rs14235	rs14235	*BCKDK*	Synonymous variant	1 (1)	TaqMan
rs14235	rs749671	*ZNF646*	Synonymous variant	0.8 (0.9)	TaqMan
rs199347	rs199355	*GPNMB*	Synonymous variant	0.93 (0.98)	TaqMan
rs823118	rs708723	*RAB7L1*	3′-UTR	0.95 (0.98)	TaqMan
rs823118	rs951366	*NUCKS1*	3′-UTR	0.72 (-0.98)	KASP
rs1474055	rs76179989	*STK39*	5′-UTR	0.94 (0.99)	TaqMan
rs8118008	rs58241213	*LZTS3*	5′-UTR	0.77 (0.89)	KASP
rs10799596	rs4649383	*SIPA1L2*	Synonymous variant	0.64 (-0.99)	KASP
rs11158026	rs28481699	*WDHD1*	3′-UTR	0.71 (0.86)	TaqMan
rs12637471	rs2270968	*MCCC1*	Missense variant	0.71 (− 1)	TaqMan
rs34311866	rs34311866	*TMEM175*	Missense variant	1 (1)	TaqMan
rs34884217	rs34884217	*TMEM175*	Missense variant	1 (1)	TaqMan
rs35749011	rs2230288	*GBA*	Missense variant	0.69 (0.83)	KASP

*SNP* single nucleotide polymorphism, *3′UTR* three prime untranslated region, *KASP* Kompetitive Allele Specific PCR.

### Validation of genotyping assays as a sensitive method for determining allelic expression

**Figure 2:**
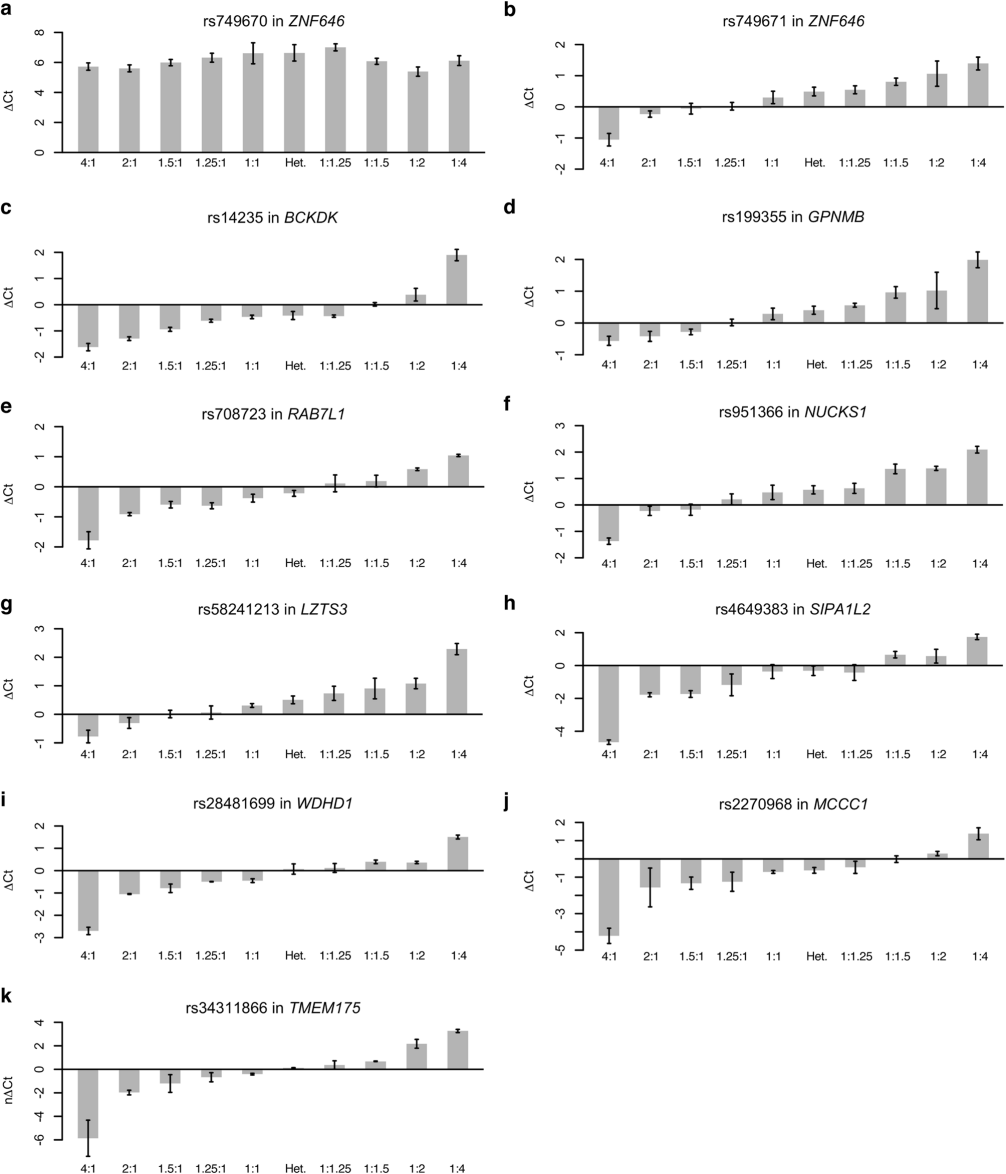
Sensitivity assessment of genotyping assays measuring allelic expression with known ratios of the alleles. Homozygous samples of genomic DNA mixtures at known ratios were tested for (**a**) rs749670 in *ZNF646*, (**b**) rs749671 in *ZNF646*, (**c**) rs14235 in *BCKDK*, (**d**) rs199355 in *GPNMB*, (**e**) rs708723 in *RAB7L1*, (**f**) rs951366 in *NUCKS1*, (**g**) rs58241213 in *LZTS3*, (**h**) rs4649383 in *SIPA1L2*, (**i**) rs28481699 in *WDHD1*, (**j**) rs2270968 in *MCCC1*, and (**k**) rs34311866 in *TMEM175* using real-time qPCR. A heterozygous (Het.) sample was included as a 1:1 ratio control as well as allele ratios 4:1, 2:1, 1.5:1, 1.25:1, 1:1, 1:1.25, 1:1.5, 1:2, and 1:4. Three assays (for SNPs rs34884217 in *TMEM175*, rs2230288 in *GBA*, and rs76179989 in *STK39*) could not be tested due to a lack of homozygous samples for one of the alleles. Data are presented as mean change in Ct between the two alleles (ΔCt). Samples were run in triplicates and error bars show the standard error of the mean. *qPCR* quantitative PCR, *Ct* cycle threshold.

We initially set out to test whether the standard genotyping assays from KASP (LGC Biosearch Technologies) and TaqMan (Applied Biosystems) were sensitive enough to identify small changes in allelic input. To achieve this, we tested the assays using samples that consisted of a mixture of gDNA from the two homozygotes for each SNP combined at nine fixed ratios (4:1, 2:1, 1.5:1, 1.25:1, 1:1, 1:1.25, 1:1.5, 1:2, and 1:4). A heterozygous gDNA sample for each SNP was included in each assay as a control for a 1:1 ratio of the alleles. One KASP assay for rs749670 in *ZNF646* did not show the expected distribution of the ratios of allele-specific signals and was excluded from further analysis (Fig. 2a). The assay was replaced by a TaqMan assay for rs749671 in *ZNF646*. All remaining tested assays showed a clear distribution of the alleles at mixed ratios (Fig. 2b–k). These results demonstrate that real-time qPCR is sufficiently sensitive to detect subtle differences in allelic ratio and so is a robust method to determine allele-specific expression.

### Identifying allele-specific expression at Parkinson’s disease GWAS risk loci in human brain tissue

To test whether a *cis*-acting PD-associated SNP affects transcript levels, we measured the relative allelic expression levels in heterozygous samples by determining the ratio of transcripts for each allele. RNA and gDNA was available from 101 human frontal cortex tissue samples from 37 PD patients and 64 age-matched controls. First, every selected transcribed SNP was genotyped, and the heterozygous samples were further analysed for allele-specific expression by quantification of the alleles in cDNA. The cDNA ratio was then normalized to the mean gDNA allelic ratio as gDNA from the heterozygous donors determine the difference between signals when the alleles are equally represented^18^.

**Figure 3:**
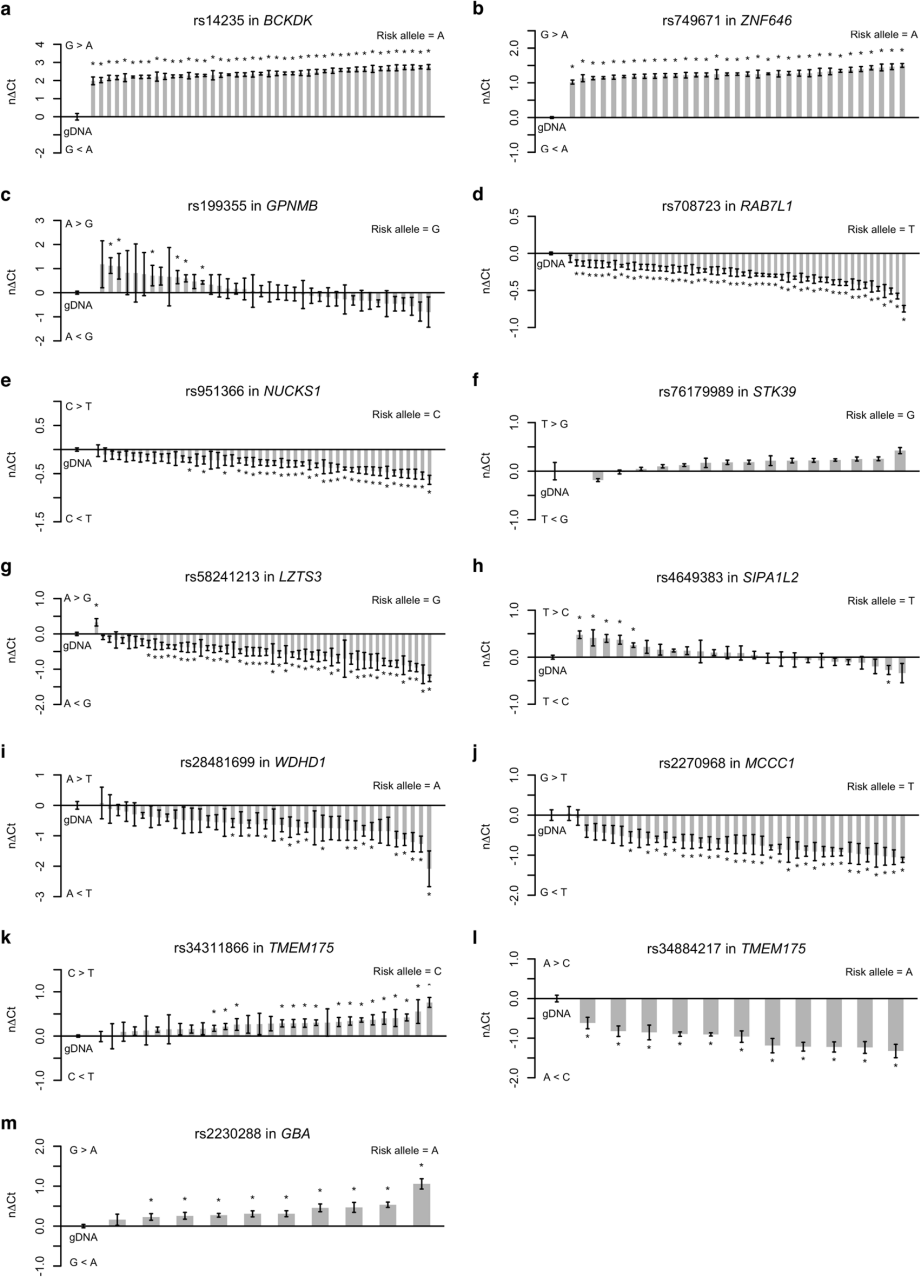
Allelic expression analyses of PD-associated risk loci in human brain samples. Allelic expression profiling was performed in heterozygous samples for (**a**) rs14235 in *BCKDK*, (**b**) rs749671 in *ZNF646*, (**c**) rs199355 in *GPNMB*, (**d**) rs708723 in *RAB7L1*, (**e**) rs951366 in *NUCKS1*, (**f**) rs76179989 in *STK39*, (**g**) rs58241213 in *LZTS3*, (**h**) rs4649383 in *SIPA1L2*, (**i**) rs28481699 in *WDHD1*, (**j**) rs2270968 in *MCCC1*, (**k**) rs34311866 in *TMEM175*, (**l**) rs34884217 in *TMEM175*, and (**m**) rs2230288 in *GBA* to determine the ratio of transcript levels for each allele. Data are presented as normalized change in Ct between the two alleles (nΔCt). Each column represents five replicate measurements and error bars show the standard error of the mean. Significant differences between each column and the genomic DNA (gDNA) control were identified using a Mann–Whitney U test. Significance was defined as *P* ≤ 0.05 symbolized with an asterisk. *Ct* cycle threshold.

The results of the allelic expression analysis for all selected PD-associated loci are summarized in Table 2, including the total number of heterozygous samples available in each assay and the overall *P* value per assay when testing for differences between all cDNA samples pooled against all gDNA samples on a global level. Among the 13 transcribed SNPs studied in 12 human genes, we observed significant allele-specific expression in 10 SNPs (Fig. 3a,b,d,e,g,i,j,k,l,m). The assay for rs58241213 in *LZTS3* (Fig. 3g) displayed a single significant outlier. In the other nine assays, we observed a consistent imbalanced expression level of one allele compared to the other in all brain samples. Importantly, the mean difference in allelic expression in cDNA compared with gDNA for each of the ten assays was highly significant (*P* < 10^–5^). For three out of 10 assays with allele-specific expression, the risk allele was more expressed than the alternative allele, whereas the other seven assays showed the opposite trend. However, there were no differences when comparing the results for PD patients and controls, indicating that the observed allele-specific expression is independent of disease status. For three SNPs, rs4649383 in *SIPA1L2*, rs199355 in *GPNMB*, and rs76179989 in *STK39* (Fig. 3c,f,h), there was no clear allele-specific expression observed. Our allelic expression analyses provide evidence that the majority of these 13 PD-associated risk loci exert their effects through *cis*-regulatory mechanisms.

**Table 2:** Allelic expression results for 12 Parkinson’s disease genome-wide associated genes in human brain samples.

Transcribed SNP	Gene	Het. samples	Risk allele	Observed ASE	Overall *P* value
rs14235	*BCKDK*	43	A	G>A	1.1 × 10^–15^
rs749671	*ZNF646*	33	A	G>A	2.2 × 10^–16^
rs199355	*GPNMB*	40	G	A=G	0.8
rs708723	*RAB7L1*	53	T	C<T	2.2 × 10^–16^
rs951366	*NUCKS1*	48	C	C<T	2.6 × 10^–12^
rs76179989	*STK39*	15	G	T=G	0.8
rs58241213	*LZTS3*	52	G	A<G	4.4 × 10^–15^
rs4649383	*SIPA1L2*	25	T	T=C	0.5
rs28481699	*WDHD1*	41	A	A<T	1.9 × 10^–6^
rs2270968	*MCCC1*	39	T	G<T	8.3 × 10^–8^
rs34311866	*TMEM175*	30	C	C>T	5.9 × 10^–7^
rs34884217	*TMEM175*	11	A	A<C	7.8 × 10^–8^
rs2230288	*GBA*	10	A	G>A	5.0 × 10^–5^

The overall *P* value was calculated using a non-parametric Mann–Whitney U test comparing the allelic expression of all genomic DNA samples with all cDNA samples.

*SNP* single nucleotide polymorphism, *Het* heterozygous, *ASE* allele-specific expression.

We selected a single locus, *GCH1/WDHD1*, from the 10 loci that displayed allele-specific expression to take forward for a preliminary examination of the regulatory mechanism using the UCSC Genome Browser^21,22^. The *GCH1/WDHD1* locus contains the lead SNP rs11158026 located in the intron of *GCH1* (Supplementary Fig. S2a) and the proxy SNP rs28481699 (r^2^ = 0.71) in the 3′-UTR of *WDHD1* for which we showed allele-specific expression (Fig. 3i). There are 53 additional SNPs in LD (r^2^ ≥ 0.6) with rs11158026, although only one has an r^2^ value over 0.9, corresponding to the proxy SNP rs3783640 that is also found in the intron of *GCH1*. rs3783640 is located in a region of closed chromatin across multiple brain regions and without regulatory element annotations in the UCSC Genome Browser^21,22^. On the other hand, data from the ENCODE Registry of Candidate *cis*-Regulatory Elements (cCREs) in the human genome^23^ suggests the lead SNP rs11158026 lies within a predicted distal enhancer element (Supplementary Fig. S2b, ENCODE accession ‘EH38E1715755′). Moreover, publicly available ATAC-seq data^24^ from neuronal cells from cortex brain regions reveal accessible chromatin peaks surrounding lead SNP rs11158026 that are not present in non-neuronal cells, making rs11158026 a more likely candidate for *cis*-regulation of *WDHD1*.

### Selected PD-associated loci are brain-specific eQTLs

**Figure 4:**
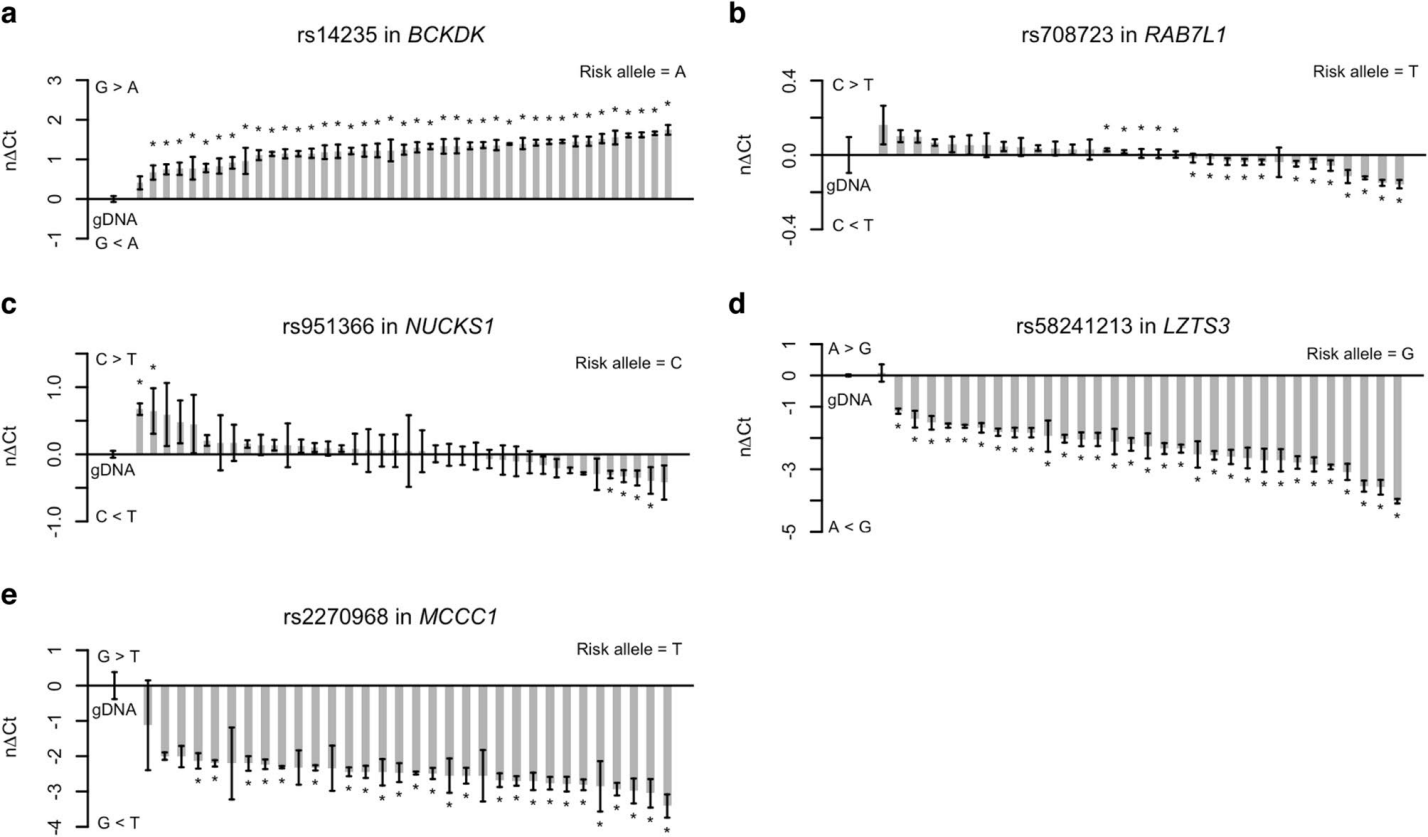
Tissue-specific allelic expression is observed in selected PD-associated risk loci. Allelic expression analysis of (**a**) rs14235 in *BCKDK*, (**b**) rs708723 in *RAB7L1*, (**c**) rs951366 in *NUCKS1*, (**d**) rs58231213 in *LZTS3*, and (**e**) rs2270968 in *MCCC1* was performed in samples from whole blood from PD patients and healthy controls. Heterozygous cDNA samples were measured with five replicates and normalized to the corresponding genomic DNA (gDNA) samples. Columns are presented as the normalized change in Ct between the two alleles (nΔCt). Error bars represent the standard error of the mean. Differences between each sample to the gDNA pool were tested with a Mann–Whitney U test and were considered statistically significant when *P* ≤ 0.05 as indicated by asterisks. *Ct* cycle threshold.

Next, we investigated whether the effects of the *cis*-regulatory mechanisms we observed through allele-specific expression in brain samples were tissue-specific. We repeated five allelic expression assays in cDNA generated from whole blood. Blood samples were collected from 54 PD patients and 40 healthy controls and genotyped for the SNPs. The five assays were chosen based on the minor allele frequency (MAF) of the PD-associated SNPs to ensure a high number of heterozygous samples. The results are summarized in Table 3. All five selected SNPs displayed allele-specific expression in brain samples. For three out of five assays, rs14235 in *BCKDK*, rs58241213 in *LZTS3*, and rs2270968 in *MCCC1* (Fig. 4a,d,e), the allele-specific expression in whole blood was consistent with our observations in the brain samples. The overall significance of differences in allelic expression between all cDNA samples compared with all gDNA samples were *P* < 10^–13^ in the three assays with allele-specific expression (Table 3). For the remaining two assays, rs708723 in *RAB7L1* and rs951366 in *NUCKS1* (Fig. 4b,c), we did not observe allele-specific expression in whole blood and the effect appears to be brain-specific.

**Table 3:** Allelic expression analysis results for five Parkinson’s disease-associated genes in blood samples.

Transcribed SNP	Gene	MAF	Het. samples	Risk allele	Observed ASE	Overall *P *value
rs14235	*BCKDK*	0.40	41	A	G>A	4.1 × 10^–16^
rs708723	*RAB7L1*	0.53	31	T	C=T	0.99
rs951366	*NUCKS1*	0.40	40	T	C=T	0.86
rs58241213	*LZTS3*	0.39	32	G	A<G	2.2 × 10^–16^
rs2270968	*MCCC1*	0.72	31	T	G<T	3.7 × 10^–13^

The overall *P* value was calculated using a non-parametric Mann–Whitney U test comparing the allelic expression of all genomic DNA samples with all cDNA samples.

*SNP* single nucleotide polymorphism, *MAF* minor allele frequency, *Het* heterozygous, *ASE* allele-specific expression.

## Discussion

The GWAS era, where thousands of genetic associations have been identified without a known causal mechanism, has created an enormous potential for understanding and treating human disease. Studies that reveal regulatory mechanisms contributing to individual diseases give considerable possibilities for improving human health by enabling the development of new diagnostic markers and novel therapeutics. Current evidence suggests that *cis*-regulatory SNPs have causal roles in many complex human diseases^25,26^. In PD, previous studies have found a non-random distribution of risk SNPs overlapping with tissue-specific putative regulatory elements^27^. Given this background, we report in the present study that the majority of the PD genome-wide associated loci examined in prefrontal cortex tissue from patients and controls had allele-specific expression effects, emphasizing the importance of investigating variation in *cis*-regulation of gene expression in disease-relevant tissue. For a small subset of genes, we also identified allele-specific effects in peripheral blood cells, raising the possibility for their use as biomarkers of PD risk.

Allele-specific expression is often associated with subtle differences in transcript levels^28^, likely a consequence of genetic and epigenetic variation in *cis*-regulatory DNA regions. To determine the precise molecular mechanisms underlying the allele-specific expression in all of the studied GWAS loci was beyond the scope of this paper, thus the interpretation of our findings remains open to discussion. A genetic interpretation of the results could involve DNA sequence variants that affect *cis*-regulatory elements, such as changes to transcription factor binding sites or microRNA binding sites resulting in altered transcription efficiency or transcript stability. Also, the allele-specific expression observed in our dataset could have an epigenetic basis, such as allele-specific methylation of DNA or allele-specific histone modifications^29,30^.

The allele-specific expression of PD GWAS-associated genes provide evidence for a number of candidate causal genes for future follow-up studies. Viewing our results in the context of already published pathway analyses can help prioritize candidate causal genes for functional studies. The results presented here are in agreement with several large-scale pathway analyses that highlight the involvement of molecular processes leading to dysregulation in mitochondrial homeostasis and lysosomal dysfunction as main contributors to PD etiology^6,31–33^. We find it interesting that two of the variants with the highest mean fold changes, rs14235 in *BCKDK* and rs2270968 in *MCCC1*, are located within genes involved in mitochondrial function. Mitochondrial impairment is a well-established pathological pathway in PD and a shared feature between the monogenic and sporadic forms of the disease^34^. Furthermore, multiple of the 24 loci identified in the meta-analysis of PD GWAS from 2014^5^ include genes that are involved in the functions of the autophagy-lysosome-pathway^35^. In line with our results, a previous study observed allele-specific expression of coding variants in two lysosomal genes, *GBA* and *TMEM175*^36^*.* These variants are non-synonymous and were top-hit SNPs in GWAS so are believed to be the functional variants. The allele-specific effect was particularly strong for rs34884217, the secondary signal in *TMEM175*. *TMEM175* encodes a lysosomal potassium channel and has been linked to the dysfunction of glucocerebrosidase and accumulation of α-synuclein^36^. The rs34884217 variant encodes a Q65P amino acid substitution in the TMEM175 protein, which is predicted to be benign and without effects on TMEM175 protein function. However, there are multiple *TMEM175* transcripts and rs34884217 is located in the first codon of exon 4, a potential splice acceptor site. The rs34884217 variant disrupts this potential splice site (AG to CG) and may affect how the *TMEM175* transcript is spliced, leading to altered expression of the *TMEM175* isoforms (Supplementary Fig. S3). We also showed allele-specific expression in the *GCH1/WDHD1* locus and found preliminary evidence for a mechanistic interpretation of our results. The lead SNP in this locus, rs11158026, is located the intron of *GCH1*. The *GCH1* gene encodes GTP cyclohydrolase 1; an essential enzyme for dopamine production in nigrostriatal cells^37^. We found that rs11158026 is located in a genomic region marked by open chromatin and a predicted enhancer element (Supplementary Fig. S2). Although further studies are warranted to confirm the true causal variant in this locus, our results are in agreement with these reports. As we have shown that allele-specific expression occurs in *WDHD1*, one interpretation is that lead SNP rs11158026 results in an altered enhancer effect influencing one or more genes, not excluding a similar enhancer effect on *GCH1* expression levels.

To analyse the allele-specific effects, we used the gDNA allelic ratio as a reliable internal control for transcript imbalance. Moreover, by examining the expression levels of alleles within the same biological sample, we have removed potential variation introduced by the environment^38^. It should also be emphasized that analysing allelic expression using real-time qPCR produced highly significant data even with a limited sample size, an important restriction when working with brain disorders. Real-time qPCR removes the presence of confounding known and unknown *trans*-acting factors, which would have added significant variation to the expression measurements. Interestingly, our study identified novel eQTLs that are not listed in the GTEx database (https://gtexportal.org/home/, last accessed November 2019). Data in the GTEx database was generated by RNA-seq, a common method for allelic expression analysis. The difference in sensitivity between the two tools, real-time qPCR and RNA-seq, could explain why our results were not in agreement with data from the GTEx database. Furthermore, it highlights the need for studies such as ours, as valuable genetic information may be overlooked when relying solely on publicly available databases.

Mendelian randomization can be used to nominate likely causal gene candidates, taking advantage of eQTL data in bination with GWAS summary statistics. The largest meta-analysis of PD GWAS to date^6^ included a Mendelian randomization analysis where the expression or methylation of 151 (64%) of 237 genes was significantly associated with a possible causal change in PD. Notably, most loci tested contained multiple putatively causal genes. In our analysis, two genes within the PARK16 locus (rs708723 in *RAB7L1* and rs951366 in *NUCKS1*) were tested and both showed allele-specific expression. In such cases, we cannot pinpoint a single gene, nor can we exclude the involvement of both genes in disease susceptibility. Thus, while allelic expression analysis has the potential to prioritize gene candidates in association regions containing more than one candidate gene, the method cannot separate between multiple genes when they all show allele-specific expression effects. Interestingly, although our data largely support the results of the Mendelian randomization analysis by Nalls et al.^6^, there are some notable differences. First, we included transcribed proxy SNPs from *GBA*, *BCKDK*, *RAB7L1* and *LZTS3*, however, these genes were not included in the Mendelian randomization analysis. Also, whereas Nalls et al. report that expression changes in *GPNMB* is associated with PD-susceptibility^6^, we did not detect any allele-specific expression effects for this gene in our brain samples. Moreover, as previously discussed, we found that *WDHD1* is an eQTL with the potential causal SNP being located in *GCH1*. Nalls et al. do not find significant changes in *WDHD1* expression in their brain or blood samples. However, the expression of *GCH1* was significantly associated with a possible causal change in PD^6^, supporting our hypothesis that the putative causal variant is located in this gene. These discrepancies may reflect the different sources of data as the Mendelian randomization analysis^6^ used expression summary statistics from GTEx, derived from RNA-seq, whereas our allelic expression analysis was generated by real-time qPCR.

The biological impact of common functional variation is cell context dependent^39^, and SNPs that are preferentially expressed in disease-relevant cell types contribute more to risk^40^. Therefore, as many traits manifest themselves only in certain tissues and cells, it is important to integrate data from the tissue of interest for the studied disease when interpreting GWAS results using gene expression as an intermediate phenotype. Neurological disorders are particularly challenging to study as brain tissue samples need to be collected post-mortem and contain a large degree of cellular heterogeneity^41^. Consequently, the choice of brain cell type is highly relevant when studying PD. It is reported that genes near PD-associated risk variants are enriched for expression in the brain, specifically in neurons from the *substantia nigra pars compacta* and the frontal cortex among others^6^. We therefore included tissue samples obtained from the medial frontal gyrus, a brain region also known to be affected by α-synuclein pathology in advanced stages of PD^42^. Furthermore, the importance of tissue choice when studying eQTLs was highlighted in a study from Hernandez and colleagues, specifically addressing whether the use of blood versus brain tissue would differ for brain phenotypes^1^. They demonstrated that while some eQTLs were shared between blood and brain tissues, there were a number of examples where brain tissue was required for eQTL discovery. This correlates well with our findings, as three out of five genes showed allele-specific expression in both blood and brain tissue and two genes were brain-specific.

A major task in the post-GWAS era is to identify the disease-conferring risk gene within a disease-associated locus. Allelic expression analysis has identified multiple genes influenced by *cis*-regulatory SNPs^43, 44^ and our results suggest that measuring allelic expression using real-time qPCR could help prioritize functional candidate genes. Moreover, the absence of allele-specific expression weakens the evidence for the disease involvement of an examined gene. In our study, the assays for rs199355 in *GPNMB*, rs76179989 in *STK39* and rs4649393 in *SIPA1L2* did not display any allele-specific expression effects. The two former SNPs were both in very high LD (r^2^ > 0.9) with the lead SNP, and so these genes may not be directly involved in PD pathogenesis. However, it should be mentioned that only 15 brain samples were heterozygous for the *STK39* variant and available for analysis, limiting the power to detect a true difference. For *SIPA1L2*, the proxy SNP rs4649393 is only moderately correlated (r^2^ = 0.64) to the lead SNP, also affecting the statistical power. This highlights two of the limitations to this approach; the dependency on transcribed proxy SNPs that meet the LD correlation threshold requirement and the availability of a sufficient number of heterozygous samples from the tissue of interest.

Additional loci have been identified in subsequent PD GWAS meta-analyses resulting in a total of 90 independent genome-wide significant signals^4, 6^. Although the majority of these novel variants have small effect sizes, increasing the odds for PD by as little as 1.05^6^, the pipeline used in this study can be applied to highlight novel disease-associated genes. We have demonstrated that allelic expression analysis is both a time- and cost-efficient method to establish *cis*-regulatory effects present in susceptibility loci, provided that a transcribed proxy SNP and disease-relevant tissue material is available. This approach can therefore easily be applied when nominating functionally relevant genes in post-GWAS follow-up work for PD and other complex disorders.

## Methods

### DNA and RNA collection from human prefrontal cortex tissue

Post-mortem brain tissue was collected from 101 individuals and kindly received from the Netherlands Brain Bank (NBB; http://www.brainbank.nl/) and Normal Aging Brain Collection (NABCA; Amsterdam UMC—location VUMC, The Netherlands)^45^. For all donors, a written informed consent for a brain autopsy and the use of the material and clinical information for research purposes had been obtained from the donor or the next of kin^46^. Autopsy was performed using a standardized protocol by NBB (open access: www.brainbank.nl). The Medical Ethics Committee of the VU University Medical Centre, Amsterdam, approved all procedures of NBB and NABCA.

We retrieved information from medical records on symptoms and signs of PD as described by MDS criteria^47^. The inclusion criteria for the PD patients in this study were: (1) clinical diagnosis of probable or established PD according to MDS criteria^47^, (2) presence of Lewy body pathology at autopsy, and (3) cases with other major neurological or psychiatric diseases were excluded from the study. Control cases included in this study did not have any neurological or psychiatric diseases during life and their brains did not show any Lewy body, AD or vascular pathology at autopsy.

Post-mortem examination was performed by an experienced neuropathologist (Annemieke JM Rozemuller, AmsterdamUMC, location VUmc) and neuroanatomist (WvB). The presence of neuropathological hallmarks was assessed following consensus criteria for diagnosis of PD^48^, Thal amyloid-β phase, Braak stage for neurofibrillary pathology and CERAD neuritic plaque scores were determined according to the National Institute on Aging—Alzheimer’s Association guidelines^49^, and Braak and McKeith stages for α-synuclein were determined according to the Brain Net Europe guidelines^42^.

Fresh-frozen tissue blocks of the right medial frontal gyrus were included, as this region is affected in late stage PD^50^ and was available for all cases in the retrospective study. The frozen tissue blocks were sectioned using a cryostat and only grey matter was included and isolated from 37 PD patients and age-matched 64 non-demented controls. DNA and RNA isolation were performed with the AllPrep DNA/RNA/miRNA Universal Kit (Qiagen) according to the manufacturer’s instructions. DNA and RNA concentrations were quantified using the NanoDrop spectrophotometer (Thermo Fisher Scientific). The purity and integrity of all RNA samples were assessed using the Bioanalyzer 2100 instrument (Agilent Technologies, mean RIN = 8.2, standard deviation = 1.4).

### Ethical approval and consent to participate

For all donors, a written informed consent for a brain autopsy and the use of the material and clinical information for research purposes had been obtained from the donor or the next of kin^46^. Autopsy was performed using a standardized protocol defined by NBB (open access: www.brainbank.nl). The Medical Ethics Committee of the VU University Medical Centre, Amsterdam, approved all procedures of NBB and NABCA.

### DNA and RNA collection from whole blood

Blood samples were collected for DNA (EDTA tubes, Greiner Bio-One) and RNA (PAXgene RNA tubes, PreAnalytiX) isolation from 54 PD patients and 40 controls. DNA isolation was performed using the Maxwell 16 LEV Blood DNA Kit (Promega) according to the manufacturer’s instructions. RNA was isolated using the Maxwell 16 LEV Blood simplyRNA Kit (Promega) according to the manufacturer’s instructions. DNA and RNA concentrations were quantified using the NanoDrop spectrophotometer (Thermo Fisher Scientific).

### cDNA synthesis

Total complementary DNA (cDNA) was prepared using 20 ng total RNA isolated from brain and blood samples which was reverse transcribed with the High-Capacity cDNA Reverse Transcription Kit (Applied Biosystems) according to the manufacturers’ protocol in a 20 μl reaction. A negative control (no-RT) was always included to monitor genomic DNA (gDNA) contamination in our samples.

### SNP selection for allelic expression analysis

Transcribed proxy SNPs were identified from the PD GWAS meta-analysis^5^ in HaploReg v4.1 (https://pubs.broadinstitute.org/mammals/haploreg/haploreg.php, last accessed December 2019). To select appropriate markers for detecting allele-specific expression, we applied a LD correlation cut-off of r^2^ ≥ 0.6 for transcribed, linked variants and selected data from individuals of European descent.

### SNP genotyping

To identify heterozygous donor samples suitable for allelic expression measurements, genotyping of all gDNA samples from blood and brain tissues was performed using either Kompetitive Allele Specific PCR (KASP, LGC Biosearch Technologies) or TaqMan (Applied Biosystems) technology (KASP and TaqMan Genotyping assay IDs available upon request). KASP genotyping reactions were carried out using 10 ng gDNA, 2.5 μl 2 × Master Mix with low ROX (LGC Biosearch Technologies) and 0.07 μl primer probes in a 5 μl total reaction. TaqMan genotyping was carried out in a 5 μl total reaction with 10 ng genomic DNA, 2.5 μl TaqMan Genotyping Master Mix (Applied Biosystems) and 0.25 μl 20 × TaqMan assay. All genotyping reactions were performed in a MicroAmp Optical 384 well reaction plate of the ViiA7 Real-Time PCR system and analysed using the SDS program v. 2.3 (Applied Biosystems).

### Sensitivity test of genotyping assays

To assess the sensitivity of the allele-specific expression assays, samples of gDNA homozygous for both alleles of the corresponding SNPs were mixed at nine ratios: 4:1, 2:1, 1.5:1, 1.25:1, 1:1, 1:1.25, 1:1.5, 1:2, and 1:4. A heterozygous gDNA sample was included as a control of the 1:1 ratio. DNA concentrations were quantified using Qubit High Sensitivity DNA spectroscopy (Invitrogen) to ensure equal sample input. The allele-specific signals of the ratios were quantified by real-time qPCR using either KASP or TaqMan genotyping assays. For KASP assays, the mixtures of ratios were analysed with 2.5 μl 2 × Master Mix with low ROX (LGC Biosearch Technologies) and 0.07 μl primer probe mix in a 5 μl total reaction. For TaqMan assays, the sample ratios were analysed in 2.5 μl TaqMan Universal Master Mix II without UNG (Applied Biosystems) with 0.25 μl 20 × primer probe mix in a 5 μl total reaction. Samples were run in triplicates on a MicroAmp Optical 384 well plate (Applied Biosystems) on the ViiA7 Real-Time PCR system (Applied Biosystems). Data was analysed by SDS v. 2.3 (Applied Biosystems).

### Allelic expression assays

Allelic expression was evaluated in heterozygous samples by determining the ratio of transcript levels for each allele using cDNA samples, and then normalized to the allelic ratio for the corresponding gDNA samples. gDNA from the same heterozygous donors was included as a control because heterozygous gDNA samples should show a 1:1 allelic ratio and any unequal amplification of the alleles must therefore be corrected for. For each assay, cDNA from heterozygous samples was measured in five replicates. Allelic expression assays with KASP technology were done in a 5 μl total reaction with 2.5 μl 2 × Master Mix with low ROX (LGC Biosearch Technologies), 0.07 μl primer probes and 5 ng cDNA or with 10 ng gDNA. TaqMan reactions were carried out in a 5 μl total reaction with 2.5 μl TaqMan Universal Master Mix II without UNG (Applied Biosystems), 0.25 μl 20 × TaqMan assays and 5 ng cDNA or 10 ng gDNA. All assays included a negative control without DNA and a no-RT control and were performed in a MicroAmp optical 384 well plate on the ViiaA7 Real-Time PCR System. The SDS v.2.3 software (Applied Biosystems) determined the cycle thresholds (Ct) values.

### Data analysis and statistics

To analyse the allelic expression measurements of heterozygous donors, the relative allelic expression of the two alleles was calculated as delta Ct (ΔCt) = Ct (FAM) − Ct (VIC). Then, the normalized ΔCt (nΔCt) was calculated by determining the difference between the allelic ratios (ΔCt) for cDNA and the mean ΔCt of all gDNA samples^18^. Samples with Ct values > 36 were excluded from further analysis as it indicates very low gene expression levels. A preliminary Shapiro–Wilk test was used to determine whether the allelic expression measurements from cDNA and gDNA were normally distributed. This preliminary test rejected the null hypothesis for normality for a selection of the assays, therefore a non-parametric test was chosen. A Mann–Whitney U test was used to identify significant differences between the ΔCt per individual cDNA sample against the mean of all gDNA samples, and to test for overall differences in the assays by combining all cDNA samples pooled against all gDNA samples. Error bars represent the standard error of the mean. Data was analysed using R Studio v. 1.1.463. *P* values < 0.05 were considered significant.

## Supplementary information

Supplementary Information.

## Data Availability

The datasets generated during and/or analysed during the current study are available from the corresponding author on reasonable request.
