# Supplementary material for: Allele-specific expression of Parkinson’s disease susceptibility genes in human brain
Source: Sci Rep. 2021 Jan 12;11:504. doi: 10.1038/s41598-020-79990-9 (PMC7804400; doi:10.1038/s41598-020-79990-9)
Supplement: Supplementary file 1 — Supplementary Information. [file 41598_2020_79990_MOESM1_ESM.pdf]

Allele-specific expression of Parkinson's disease susceptibility genes in human brain

Margrete Langmyhr, MSc[1,2], Sandra Pilar Henriksen, MSc[1], Chiara Cappelletti, MSc[3], Wilma D. J. van de Berg, PhD[4], Lasse Pihlstrøm, MD, PhD[1], and Mathias Toft*, MD, PhD[1,2]

[1]Department of Neurology, Oslo University Hospital, Oslo, Norway

[2]Institute of Clinical Medicine, University of Oslo, Oslo, Norway

[3]Department of Mechanical, Electronic and Chemical Engineering, OsloMet – Oslo Metropolitan University, Norway

[4]Department of Anatomy and Neurosciences, Section Clinical Neuroanatomy and Biobanking, Amsterdam Neuroscience, Amsterdam UMC, location VU Medical Center, Amsterdam, the Netherlands

* Corresponding author: **mathias.toft@medisin.uio.no**

SUPPLEMENTARY MATERIAL

Supplementary Methods	Page 2
Supplementary Figure S1	Page 3
Supplementary Figure S2	Page 6
Supplementary Figure S3	Page 8
References	Page 9

Supplementary Methods

Analysis of BOCA ATAC-seq data and ENCODE data in UCSC Genome Browser

ATAC-seq data from the Brain Open Chromatin Atlas (BOCA) online database[1] (https://bendlj01.u.hpc.mssm.edu/multireg/) was loaded into the UCSC Genome Browser[2,3]. The data originates from a study[1] where they map chromatin accessibility in neuronal and non-neuronal nuclei across 14 brain regions: dorsolateral prefrontal cortex, orbitofrontal cortex, ventrolateral prefrontal cortex, anterior cingulate cortex, superior temporal cortex, inferior temporal cortex, primary motor cortex, insula, primary visual cortex, amygdala, hippocampus, mediodorsal thalamus, nucleus accumbens and putamen[1]. Briefly, they combined fluorescence-activated nuclear sorting followed by ATAC-seq on 122 nuclear samples obtained from dissections from 14 brain regions of five subjects from frozen human post-mortem tissue. The human Encyclopedia of DNA Elements (ENCODE)[4] registry of candidate *cis*-regulatory elements (cCREs)[5,6] track was loaded into the UCSC Genome Browser. cCREs are defined by representative DNase hypersensitive sites across ENCODE and Roadmap Epigenomics samples with supporting data from histone modifications (H3K4me3 and H3K27ac) and CTCF-binding. Results are presented as screen shots of human genome GRCh38/hg38 (December 2013) and GRCh37/hg19 (February 2009) assemblies in the UCSC Genome Browser (http://genome.ucsc.edu).

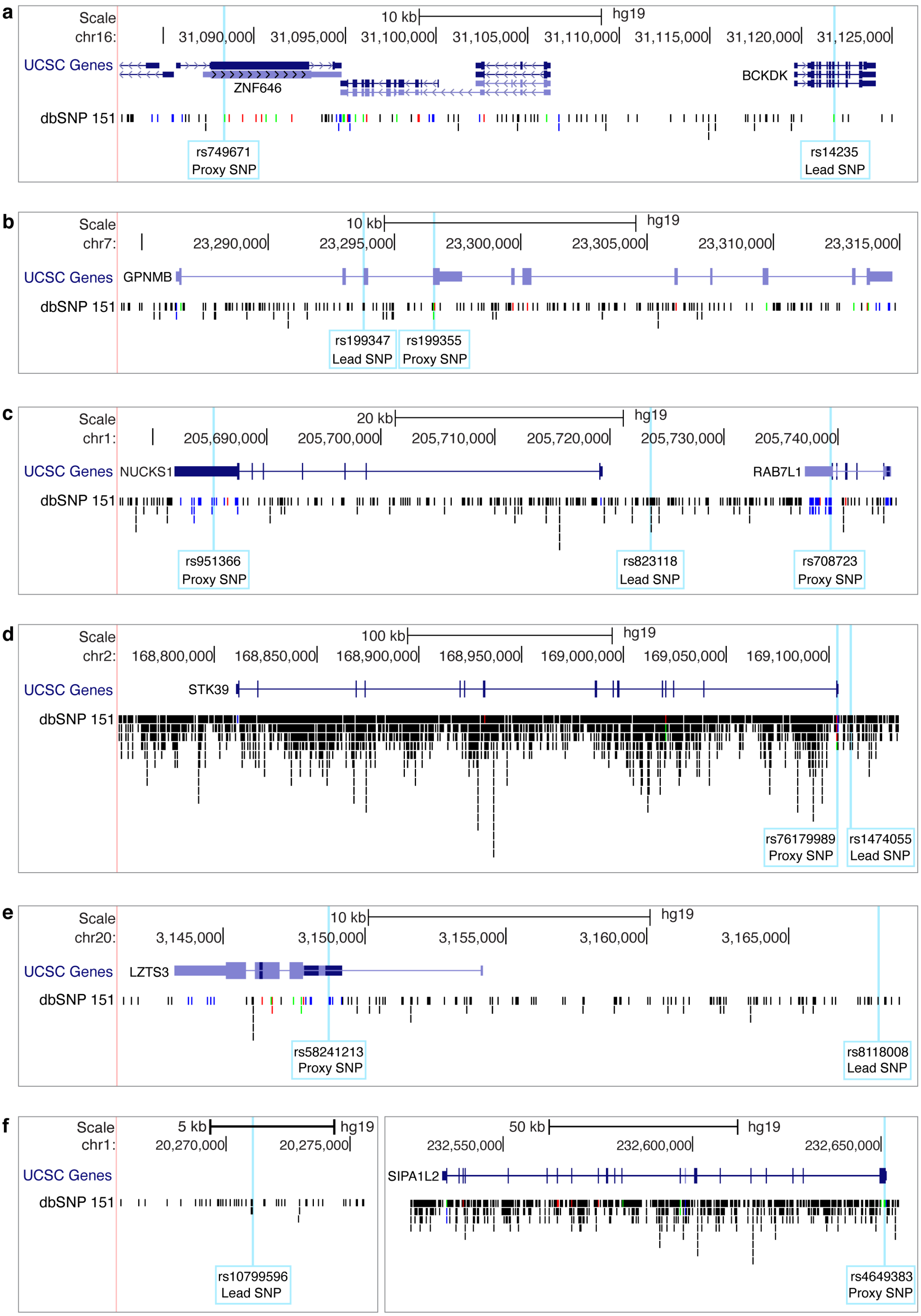
a
Scale
chr16:
10 kb
hg19
31,090,000
31,095,000
31,100,000
31,105,000
31,110,000
31,115,000
31,120,000
31,125,000
UCSC Genes
ZNF646
BCKDK
dbSNP 151
rs749671
Proxy SNP
rs14235
Lead SNP
b
Scale
chr7:
10 kb
hg19
23,290,000
23,295,000
23,300,000
23,305,000
23,310,000
23,315,000
UCSC Genes
GPNMB
dbSNP 151
rs199347
Lead SNP
rs199355
Proxy SNP
c
Scale
chr1:
20 kb
hg19
205,690,000
205,700,000
205,710,000
205,720,000
205,730,000
205,740,000
UCSC Genes
NUCKS1
RAB7L1
dbSNP 151
rs951366
Proxy SNP
rs823118
Lead SNP
rs708723
Proxy SNP
d
Scale
chr2:
100 kb
hg19
168,800,000
168,850,000
168,900,000
168,950,000
169,000,000
169,050,000
169,100,000
UCSC Genes
STK39
dbSNP 151
rs76179989
Proxy SNP
rs1474055
Lead SNP
e
Scale
chr20:
10 kb
hg19
3,145,000
3,150,000
3,155,000
3,160,000
3,165,000
UCSC Genes
LZTS3
dbSNP 151
rs58241213
Proxy SNP
rs8118008
Lead SNP
f
Scale
chr1:
5 kb
hg19
20,270,000
20,275,000
UCSC Genes
dbSNP 151
rs10799596
Lead SNP
50 kb
hg19
232,550,000
232,600,000
232,650,000
SIPA1L2
rs4649383
Proxy SNP

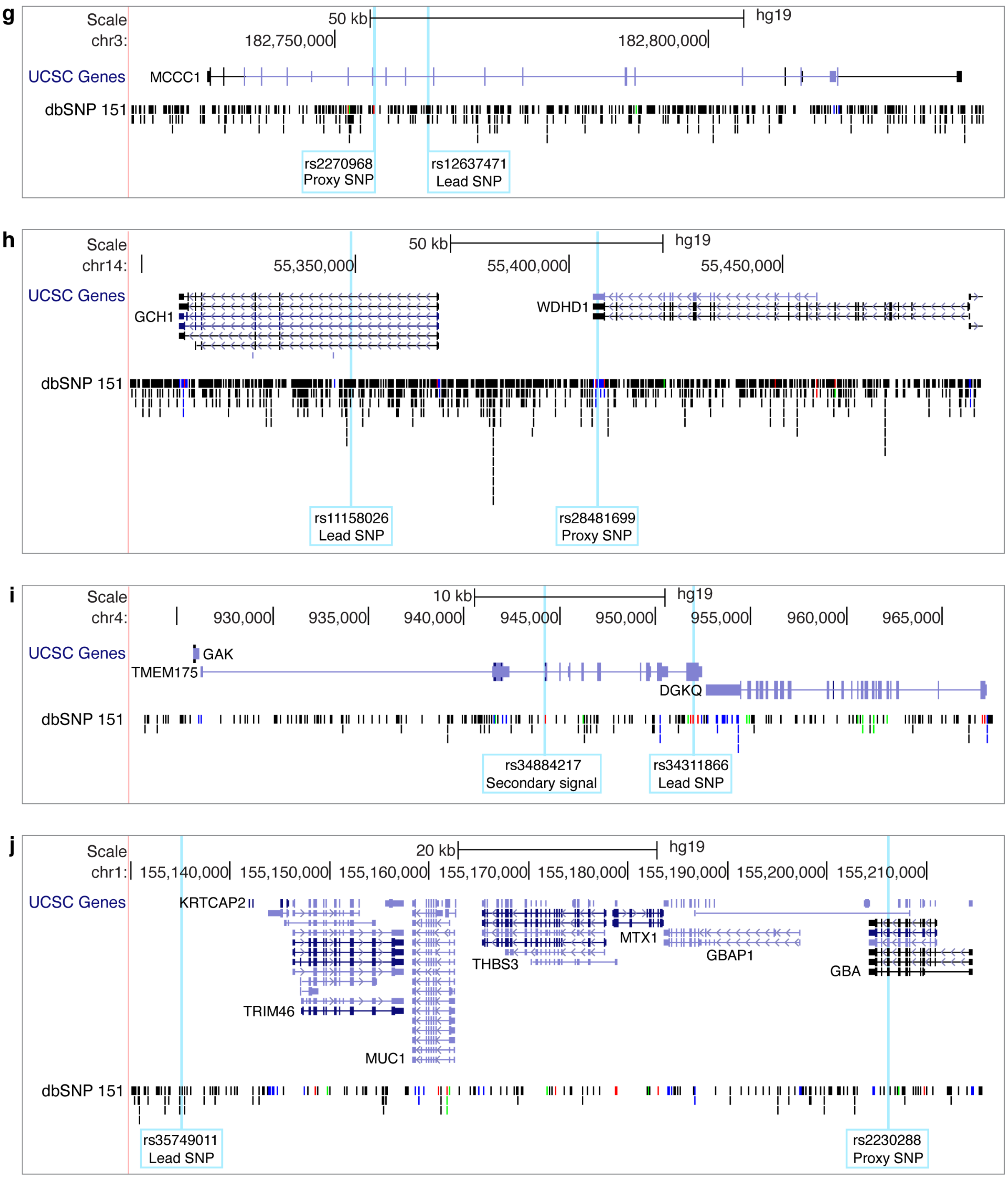

Supplementary Figure S1. **Tracks from the UCSC Genome Browser showing the genomic location of the lead and proxy SNPs in all of the analysed Parkinson's disease-associated loci in human genome assembly GRCh37/hg19.** Screen shots from UCSC Genome Browser (http://genome.ucsc.edu) of (**a**) lead SNP rs14235 in *BCKDK* and proxy SNP rs749671 in *ZNF646*, (**b**) lead SNP rs199347 and proxy SNP rs199355 in *GPNMB*, (**c**) lead SNP rs823118 and proxy SNP rs951366 in *NUCKS1* and proxy SNP rs708723 in

RAB7L1, (**d**) lead SNP rs1474055 and proxy SNP rs76179989 in *STK39*, (**e**) lead SNP rs8118008 and proxy SNP rs58241213 in *LZTS3*, (**f**) lead SNP rs10799596 and proxy SNP rs4649383 in *SIPA1L2*, (**g**) lead SNP rs12637471 and proxy SNP rs2270968 in *MCCC1*, (**h**) lead SNP rs11158026 in *GCH1* and proxy SNP rs28481699 in *WDHD1*, (**i**) lead SNP rs34311866 and secondary signal rs34884217 in *TMEM175*, (**j**) lead SNP rs35749011 and proxy SNP rs2230288 in *GBA*. Genes are demonstrated with the UCSC Genes track and SNPs are demonstrated with the dbSNP151 track[7].

SNP: Single nucleotide polymorphisms.

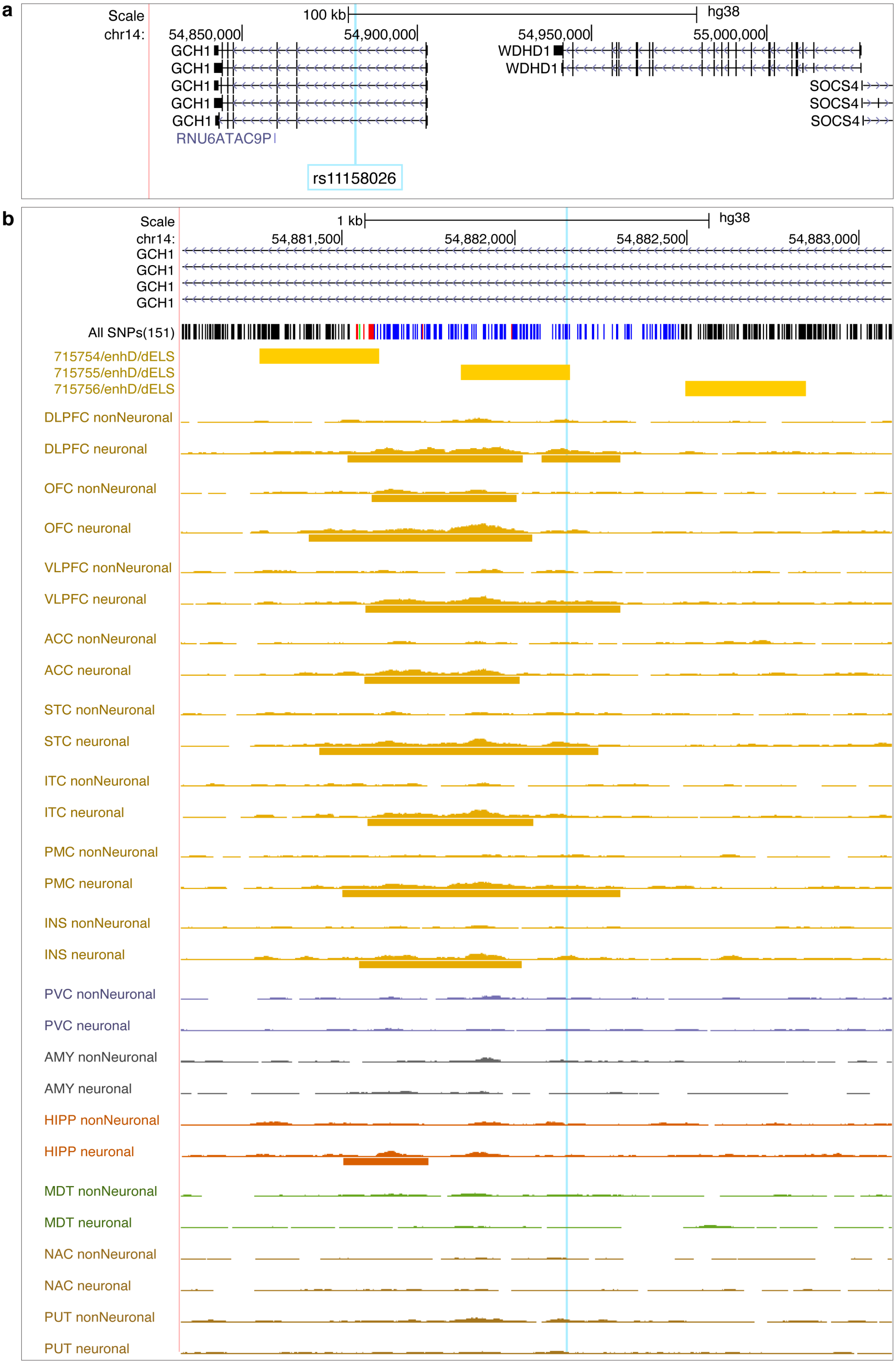
a
Scale
chr14:
100 kb
hg38
54,850,000
54,900,000
54,950,000
55,000,000
GCH1
GCH1
GCH1
GCH1
GCH1
RNU6ATAC9P
WDHD1
WDHD1
SOCS4
SOCS4
SOCS4
rs11158026
b
Scale
chr14:
1 kb
hg38
54,881,500
54,882,000
54,882,500
54,883,000
GCH1
GCH1
GCH1
GCH1
All SNPs(151)
715754/enhD/dELS
715755/enhD/dELS
715756/enhD/dELS
DLPFC nonNeuronal
DLPFC neuronal
OFC nonNeuronal
OFC neuronal
VLPFC nonNeuronal
VLPFC neuronal
ACC nonNeuronal
ACC neuronal
STC nonNeuronal
STC neuronal
ITC nonNeuronal
ITC neuronal
PMC nonNeuronal
PMC neuronal
INS nonNeuronal
INS neuronal
PVC nonNeuronal
PVC neuronal
AMY nonNeuronal
AMY neuronal
HIPP nonNeuronal
HIPP neuronal
MDT nonNeuronal
MDT neuronal
NAC nonNeuronal
NAC neuronal
PUT nonNeuronal
PUT neuronal

Supplementary Figure S2. The *GCH1/WDHD1* locus in the UCSC Genome Browser. (**a**) Screen shot of the *GCH1/WDHD1* locus in the UCSC Genome Browser (http://genome.ucsc.edu) in human genome assembly GRCh38/hg38, highlighting the position of lead SNP rs11158036 in the first intron of *GCH1*. (**b**) Lead SNP rs11158026 in *GCH1* (blue vertical line) is located in the predicted enhancer element '715755/enhD/dELS' in the ENCODE Registry of Candidate *cis*-Regulatory Elements (cCREs) (top tracks)[6]. ATAC-seq data from neuron and non-neuron cells across 14 distinct brain regions was downloaded from BOCA (bottom tracks)[1]. The rs11158026 SNP is located in neuronal-specific open chromatin in the DLPFC, VLPFC, STC and PMC brain regions.

SNP: single nucleotide polymorphism, ATAC-seq: Assay for Transposase-Accessible Chromatin with high-throughput sequencing data, BOCA: Brain Open Chromatin Atlas, DLPFC: Dorsolateral prefrontal cortex, OFC: Orbitofrontal cortex, VLPFC: Ventrolatelar prefrontal cortex, ACC: Anterior cingulate cortex, STC: Superior temporal cortex, ITC: Inferior temporal cortex, PMC: Primary motor cortex, INS: Insula, PVC: Primary visual cortex, AMY: Amygdala, HIP: Hippocampus, MDT: Mediodorsal thalamus, NAC: Nucleus accumbens, PUT: Putamen.

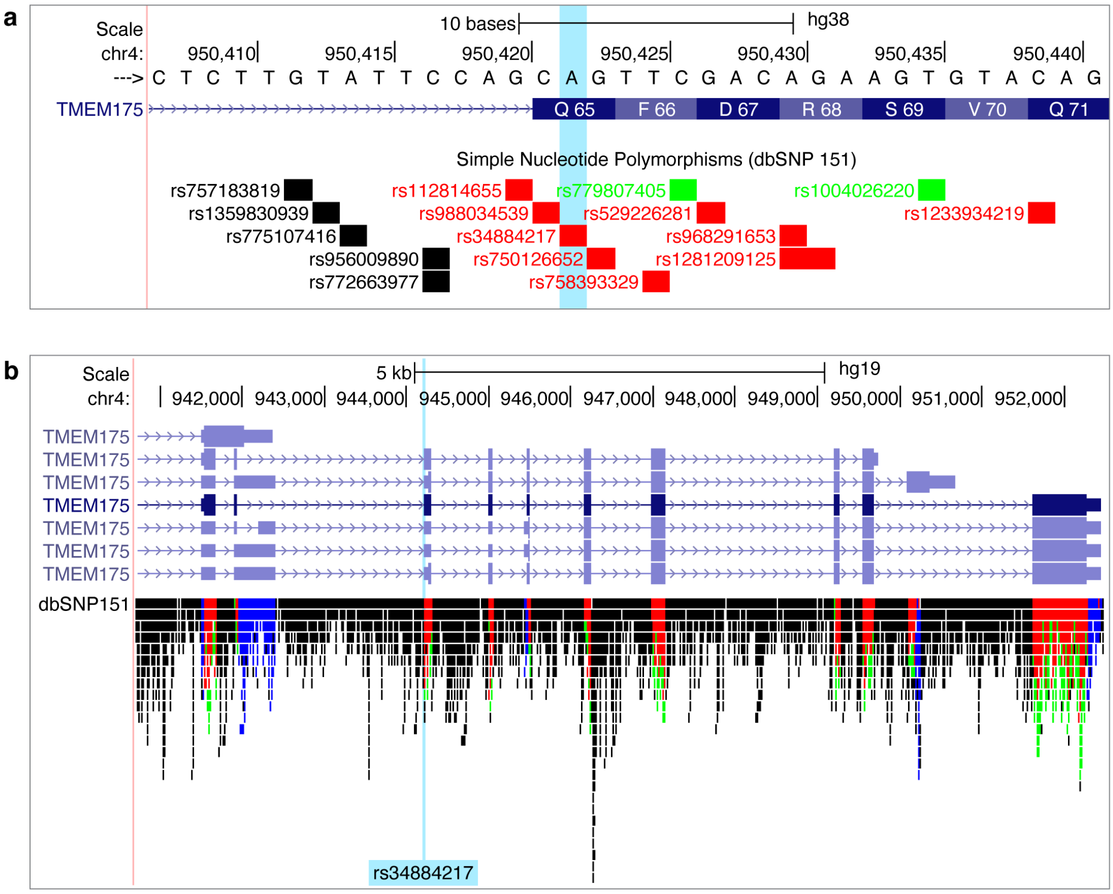

Supplementary Figure S3. **The *TMEM175* locus**. Screen shot of the UCSC Genome Browser (http://genome.ucsc.edu) showing the location of (**a**) secondary signal rs34884217 in the *TMEM175* locus in a putative alternative splice site (AG to CG) in human genome assembly GRCh38/hg38 and (**b**) the alternatively spliced isoforms of *TMEM175* in human genome assembly GRCh37/hg19. Genes are demonstrated with the UCSC Genes track and SNPs are demonstrated with the dbSNP151 track[7].

References

1. Fullard, J.F. et al. An atlas of chromatin accessibility in the adult human brain. *Genome Res* **28**, 1243-1252 (2018).
2. Haeussler, M. et al. The UCSC Genome Browser database: 2019 update. *Nucleic Acids Res* **47**, D853-d858 (2019).
3. Kent, W.J. et al. The human genome browser at UCSC. *Genome Res* **12**, 996-1006 (2002).
4. Rosenbloom, K.R. et al. ENCODE data in the UCSC Genome Browser: year 5 update. *Nucleic Acids Res* **41**, D56-63 (2013).
5. ENCODE Project Consortium. An integrated encyclopedia of DNA elements in the human genome. *Nature* **489**, 57-74 (2012).
6. Moore, J.E. et al. Expanded encyclopaedias of DNA elements in the human and mouse genomes. *Nature* **583**, 699-710 (2020).
7. Sherry, S.T. et al. dbSNP: the NCBI database of genetic variation. *Nucleic Acids Res* **29**, 308-11 (2001).
